# Chinese Populations of *Magnaporthe oryzae* Serving as a Source of Human-Mediated Gene Flow to Asian Countries: A Population Genomic Analysis

**DOI:** 10.3390/jof10110739

**Published:** 2024-10-25

**Authors:** Guohua Duan, Yuchan Liu, Cheng Zheng, Kaihui Yu, Jiahui Xie, Baohua Wang, Huakun Zheng, Wei Tang, Jiandong Bao, Zonghua Wang, Meilian Chen

**Affiliations:** 1 College of Materials and Chemical Engineering, Fujian Key Laboratory on Conservation and Sustainable Utilization of Marine Biodiversity, Minjiang University, Fuzhou 350108, China; ghduan1990@163.com (G.D.); zczcdf@126.com (C.Z.); kaihui1107@163.com (K.Y.); 2 College of Plant Protection, Jilin Provincial Key Laboratory of Green Management of Crop Pests and Diseases, Jilin Agricultural University, Changchun 130118, China; yuchan_liu@163.com; 3 Jilin Institute of Chinese Engineering Development Strategies, Changchun 130118, China; 4 State Key Laboratory of Ecological Pest Control for Fujian and Taiwan Crops, College of Plant Protection, Fujian Agriculture and Forestry University, Fuzhou 350002, China; bbxjh1994@163.com (J.X.); wbaohua@fafu.edu.cn (B.W.); tangw@fafu.edu.cn (W.T.); baojd@zaas.ac.cn (J.B.); 5 Fujian Universities Key Laboratory for Plant Microbe Interaction, College of Life Sciences, Fujian Agriculture and Forestry University, Fuzhou 350002, China; huakunzheng@163.com

**Keywords:** *Magnaporthe oryzae*, population structure, genetic diversity, gene flow

## Abstract

*Magnaporthe oryzae*, a filamentous heterothallic ascomycete fungus that serves as the causative agent of rice blast disease, is globally distributed in rice-growing regions. Populations shaped by environmental factors and human intervention play important roles in the formation of genetic structure. In this study, population structures and spatiotemporal dynamics were investigated based on large-scale whole genomic sequences of rice-infecting *M. oryzae* around the world. By analyzing these genetic structures, we identified divergent clades that crossed geographic boundaries. While we observed associations between the isolates and their geographic origins, we also found that there were frequent migration events occurring across Asia in main rice cultivation regions. Within Asia, China was the migration origin, facilitating gene flows to Japan and South Korea. Since the 1970s, the genetic diversity of *M. oryzae* populations in China has also shown a steadily increasing trend, continuing through to the 2020s. Additionally, our analysis of the evolutionary history of Asian *M. oryzae* populations provided insights into the population expansion that has taken place in recent decades. Overall, our findings indicate that human-mediated gene flows played a pivotal role in shaping the genetic structure of *M. oryzae*.

## 1. Introduction

Rice blast, caused by the filamentous ascomycete fungus *Magnaporthe oryzae*, is a devastating disease that occurs in rice-growing areas worldwide and poses a serious threat to global food security [1]. *M. oryzae* can rapidly overcome resistance genes in rice and can coexist with resistant varieties within a few years of their initial deployment in rice agroecosystems [2,3]. The pathogen infects rice throughout the growth period and has a wide host range that includes more than 50 cultivated and wild monocot plants, such as rice (*Oryza sativa*), barley (*Hordeum vulgare*), wheat (*Triticum aestivum*), finger millet (*Eleusine coracana*), goosegrass (*Eleusine indica*), perennial ryegrass (*Lolium perenne*) and more [4]. Its asexual reproduction is prevalent in most rice fields, resulting in local populations of *M. oryzae* often exhibiting only one mating type [5,6,7,8,9].

It is necessary to better understand the features of the genetic structure of *M. oryzae* in order to provide the genetic basis for developing sustainable and effective prevention and control strategies for rice blast disease [10]. Genetic diversity can reflect the survival ability and adaptive potential of natural populations in the face of rapidly changing biotic and abiotic backgrounds [11]. For the plant pathogen *M. oryzae*, genetic diversity is usually estimated using molecular markers, including microsatellite markers, or DNA fingerprinting via amplified fragment length polymorphism. These methods have been applied in several countries and continents, including China [12,13,14], India [15,16], Thailand [17], the Philippines [18], Africa [19], Europe [20] and America [21], and have revealed regional diversity variations underling local adaptations [22]. The establishment of genetic structures can be driven by geographic isolation and ecological factors. In a previous population genomic analysis, three main genetic clades were identified in *M. oryzae*, which were associated with the distribution of mating types [23,24]. In a worldwide population structure analysis of *M. oryzae*, multiple endemic and pandemic lineages were identified, which were distributed in specific rice-growing areas [25]. Based on the amplified fragment length polymorphism, very few genetic differences have been found between the geographically distant populations of *M. oryzae* in Iran and those in Uruguay, although evidence of gene flow has been observed [26]. As a broad host pathogen, host specialization has also been reported to be a non-negligible factor in the genetic differentiation of *M. oryzae* [27,28]. However, the limited availability of genetic markers has hindered population genetic analysis from accurately identifying the comprehensive ensemble population structures of rice-infecting lineages due to insufficient information regarding the incomplete divergent populations. In particular, there remains a scarcity of studies addressing the spatiotemporal dynamics of genetic diversity and population structures of *M. oryzae* through large-scale genomic analysis.

Gene flow, or migration, implies the movement of genetic material among spatially or temporally separated populations and acts as a major driving force for organisms to establish population structures. It can accelerate novel variations through gene recombination, migrating to found new populations and takeovers of other local populations. Island [29] and stepping-stone models [30] have indicated that the migration of one or more subpopulations can decrease the genetic correlation with geographical distance among sexually reproducing species. For plant pathogens, spatial movement occurs in many forms, such as short-distance transfer through rainwater and long-distance global transfer, which counteracts the disadvantage of immobility for large-scale epidemics. Population structures are generally established by genetic variations and natural factors, as well as anthropogenic changes, especially among crop pathogens that are closely linked to human survival. Therefore, human movement significantly influences the migration of plant pathogens [31,32].

In agricultural ecosystems, human movement can aid the extensive spread of plant pathogens by breaking inherent mobility limitations or geographic barriers, such as rivers and mountains, that can restrict population expansion, thereby contributing to the complexity of population structures. Therefore, the globalization of agricultural products, largely driven by human activities, has led plant pathogens to evolve toward metapopulation formation [31]. For example, the transportation of infected seeds is one of that ways that *M. oryzae* has migrated, demystifying the close genetic relationships that have arisen between geographically separate populations [33]. Through the genome sequence analysis of global *M. oryzae* populations over different time periods, a sexual recombination signature was detected in the Southeast Asian endemic lineage, suggesting the occurrence of gene flows among geographic population distributions [25]. Based on microsatellite markers, *M. oryzae* has been found to have weak geographic structures in three island groups with limited natural migration, which was induced by the transportation of infected seeds around the Philippines [18]. Based on amplified fragment length polymorphism analysis, frequent gene flows have been discovered in East African populations [34], as well as between the different provinces in Korea [35]. This has also occurred with other plant viruses; for example, turnip mosaic potyvirus spread from west to east regions in Eurasia due to historical trade arteries, such as the Silk Road [36].

In this study, we first collected the published whole-genome sequences of 189 rice-infecting *M. oryzae* isolates from five continents and 22 re-sequenced genomes from China. Then, we analyzed their genetic diversity and population structure characteristics to evaluate population divergence driven by spatiotemporal changes. We found that the *M. oryzae* population in China exhibited the highest genetic diversity in Asia, which has been increasing since the 1970s, and could be divided into three divergent clades. The diffusion route of *M. oryzae* followed human activity, suggesting that the population in China served as the genetic pool for rice-growing regions across Asia, with China being the migration origin. Our study provides a detailed understanding of the spatiotemporal dynamics of genetic diversity and population structures, which could be useful for developing cultivars with different resistant genes and could contribute new insights into disease management.

## 2. Materials and Methods

### 2.1. Isolate Sampling, DNA Preparation, Genome Sequencing and Published Genome Collection

We collected 22 rice-infecting *M. oryzae* isolates from rice fields in China. The sampling locations are described in Table S1. Genomic DNA was extracted from fresh mycelium cultures after single-spore isolation, as previously described [37]. An Illumina paired-end DNA sample prep kit (Illumina, San Diego, CA, USA) was employed to construct the Illumina fragment libraries and an Illumina HiSeq2500 instrument (Novogen, Beijing, China) was used for the sequencing.

A total of 189 *M. oryzae* genomes have been sequenced from rice, which we downloaded from the NCBI database using the ncbi-genome-download script (https://github.com/kblin/ncbi-genome-download, accessed on 16 March 2023). This genome information is also listed in Table S1.

### 2.2. Identification of Single-Nucleotide Polymorphism

To obtain high-quality single-nucleotide polymorphism sites from the sequencing reads, the raw data were first trimmed using the Trimmomatic software v0.39 [38]. The eighth version genome of the strain 70-15 was taken as the reference genome and the paired-end reads were aligned against it using the BWA v0.7.17 as the default parameters [39,40]. Then, single-nucleotide polymorphism (SNP) mining was implemented using the Genome Analysis Toolkit (GATK v4.1.4.1), which compared the reference genome to the standard settings [41]. After that, VCFtools v0.1.17 was used to filter biallelic sites with minor allele counts less than 3 and missing data lower than 20% [42].

For the genomes downloaded from the NCBI database, genome-to-genome SNP identification was performed using the -maxmatch and -c 100 options in MUMMer v4.0.0 [43] against the reference genome.

### 2.3. Characterization of Genetic Diversity and Population Genetic Structures

To estimate the genetic diversity of *M. oryzae* populations that were divided by spatiotemporal boundaries, the nucleotide diversity of whole-genome SNPs was calculated using the “PopGenome” v2.1.6 package with a 20 kb sliding window and 2 kb step size [44]. The statistical significance between each pair of geographical populations was estimated using a Student’s *t*-test (two-tailed). A SNP-based neighbor-joining phylogenetic tree was reconstructed according to the bitwise distance with 1000 bootstrap replicates using the “poppr” package v2.9.3 in R v4.0.5 [45]. A principal component analysis (PCA) of the genetic structures of global populations from Asia, Europe, North America and South America was implemented on the matrix of binary allele sites using the “adegenet” package v2.1.3 [46].

To establish the population structure compositions, the SNP dataset was pruned based on linkage disequilibrium (LD) with a value of r2 = 0.5 using Plink v1.9 (https://www.cog-genomics.org/plink/1.9, accessed on 29 March 2023). To evaluate the individual ancestry components and admixture proportions, a population admixture analysis was performed using the ADMIXTURE v1.3.0 program [47]. In ADMIXTURE, 5-fold and 10-fold cross-validation procedures with K values between 2 and 10 were implemented to identify the optimal number of population clusters (K) with the lowest cross-validation error values. The admixture proportions of each sample from the geographic populations were visualized using PopHelper v2.3.0 [48].

### 2.4. Phylogeographic Analysis of M. oryzae

To detect the reticulation events of the populations, such as hybridization, horizontal gene transfer and recombination, the phylogenetic networks were reconstructed using the SplitsTree v4.14.6 [49]. In order to assess the robustness of the tree’s topology, 1000 bootstrap replicates were performed using the “Neighbor-Net” and “uncorrected p-distance” parameters.

To ascertain the phylogeographical dispersal routes (including migration routes and directionality) of *M. oryzae* across Asia, we employed the asymmetric discrete trait substitution model with the Bayesian stochastic search variable selection approach [50] using BEAST 1.10.4 [51]. Based on the Bayesian information criterion, the best nucleotide substitution model was estimated using jModeltest v2.1.2 [52] after converting the SNP dataset into the Phylip format using vcf2phylip v2.0 [53]. The posterior distributions of the parameters were estimated using two independent Markov chain Monte Carlo (MCMC) methods for 100 million generations, with sampling every 20,000 generations. Tracer v1.7 was used to check model convergence (effective sample size > 200) after excluding 10% of the initial samples as burn-in [54]. Using the SpreaD3 v0.9.6 software, a Bayes factor analysis (BF > 3) was performed to identify diffusion routes that were strongly supported by the data [55].

## 3. Results

### 3.1. Temporal Dynamics of Genetic Diversity in Chinese M. oryzae Populations

**Figure 1:**
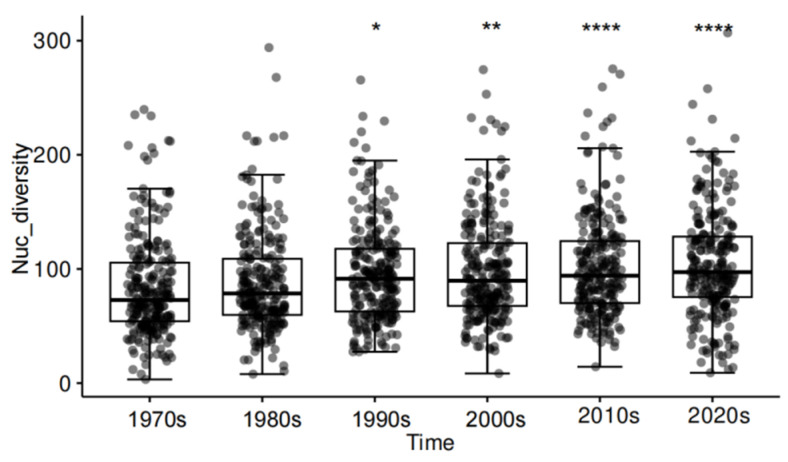
The nucleotide diversity indices for rice-infecting *M. oryzae* populations by time in China. The dynamics of nucleotide diversity were based on whole-genome SNPs with 20 kb sliding windows. The nucleotide diversity distribution in different decades from the year 1976 to 2021 is shown on the x-axis. Boxes show the first quartile, median and third quartile, and whiskers extend to 1.5 times the interquartile range. Values for each of the windows are shown as points scattered in boxplots. * *p* < 0.05, ** *p* < 0.01, **** *p* < 0.0001.

After SNP mining and filtering, a final SNP dataset of 204,626 whole-genome SNPs was obtained from the samples, which was applied for the evaluation of the genetic diversity of each decadal population of *M. oryzae*. A nucleotide diversity statistical analysis revealed variations among the populations from the 1970s to 2020s, indicating an average nucleotide diversity index (Pi) of 83.56–104.28 for each analysis window (Figure 1). Over time, the nucleotide diversity of *M. oryzae* populations has exhibited a steady upward trend. Beginning with a diversity value of 83.56 in the 1970s, this gradual increase continued through the decades: 88.10 in the 1980s, 96.09 in the 1990s, 98.04 in the 2000s, and 102.30 in the 2010s. Notably, pairwise *t*-test comparisons revealed that the nucleotide diversity of samples from the 2020s was the highest observed, with a value of 104.28.

### 3.2. Genetic Structures of Worldwide Geographic Populations

**Figure 2:**
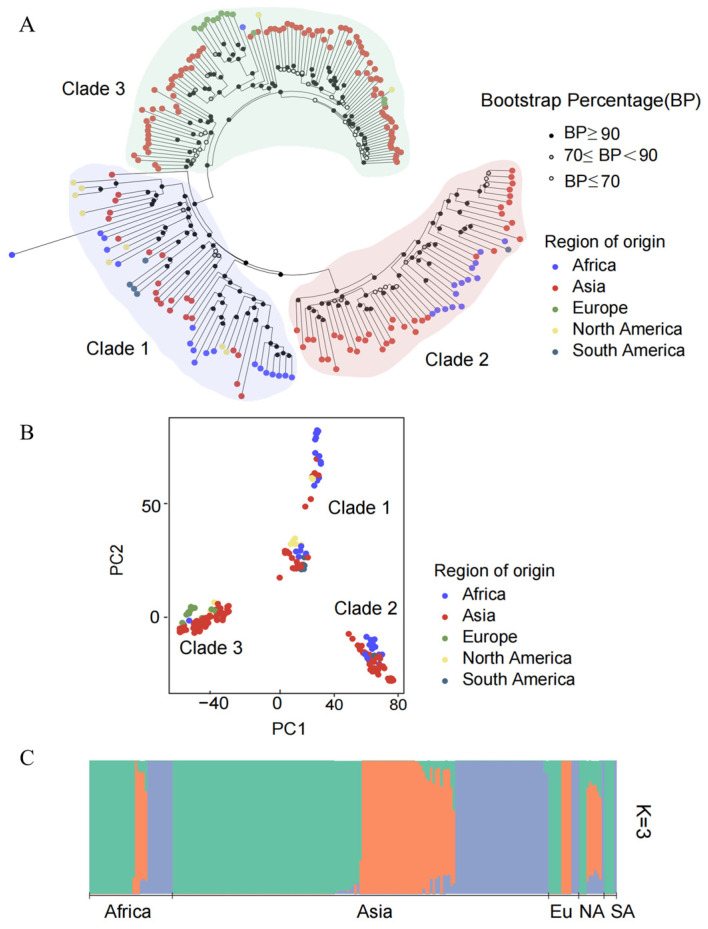
Population genetic structure analysis of *M. oryzae* with different geographical origins. (**A**) Phylogenetic tree based on whole-genome single-nucleotide genetic variant data for worldwide samples using the neighbor-joining (NJ) method with 1000 bootstrap replicates. The colored dots at the branch tips of the phylogenetic tree represent the geographical origins of the samples, while the color of the irregular shaded areas indicates the isolates included in each clade; (**B**) principal component analysis (PCA) for populations from five continents (each dot represents one individual and the colors correspond to the sample collection locations); (**C**) ADMIXTURE bar plot of the ancestral assignments of individual isolates using the optimal K  =  3 (the length of each colored segment corresponds to the proportion of the individual’s genome ancestry).

A neighbor-joining (NJ) tree was constructed for the global isolates of *M. oryzae* based on the whole-genome SNPs, which showed that the worldwide samples formed three distinct sub-clades (Figure 2A). Notably, the samples from Asia were distributed into three sub-clades. However, for the isolates from Europe, North America, South America and Africa, although there were a few isolates that were localized in other clades, the majority of the individual isolates were assigned to a single clade. Therefore, there was no obvious segregation of individuals based on geographical location. The results of the PCA corresponded to the phylogenetic tree topology, indicating that individuals were grouped into three clusters with incomplete separation among the geographic populations (Figure 2B).

Furthermore, population structure composition analysis using ADMIXTURE revealed the individual ancestry and admixture proportions within each population. The optimum cluster number was K = 3 (Table S2), as it had the lowest cross-validation error, and it was thus selected as the number of potential ancestors for worldwide *M. oryzae* populations (Figure 2C). Among them, the genetic compositions of the isolates from Asia, Africa, Europe and North America contained three ancestral lineages, although the individuals were dominated by a single ancestral lineage. However, some isolates contained two ancestral lineages. Notably, the European samples consisted of three independent ancestral lineages, without any mixed multilineage isolates. In addition, the isolates from South America were descended from two independent lineages.

### 3.3. Migration Patterns of M. oryzae

**Figure 3:**
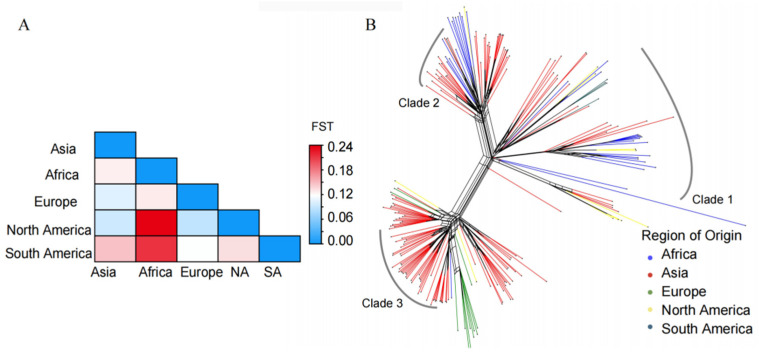
Phylogenetic network of worldwide *M. oryzae* isolates according to a Neighbor-Net analysis. (**A**) The heatmap of genetic differentiation (FST) between populations with different geographical origins. (**B**) The analysis was implemented using SplitsTree v4.14.6 with the “Neighbor-Net” and “uncorrected p-distance” parameters. The reticulate dendrogram in the tree represents incompatible signals, implying incompatible or ambiguous relationships between the samples.

**Figure 4:**
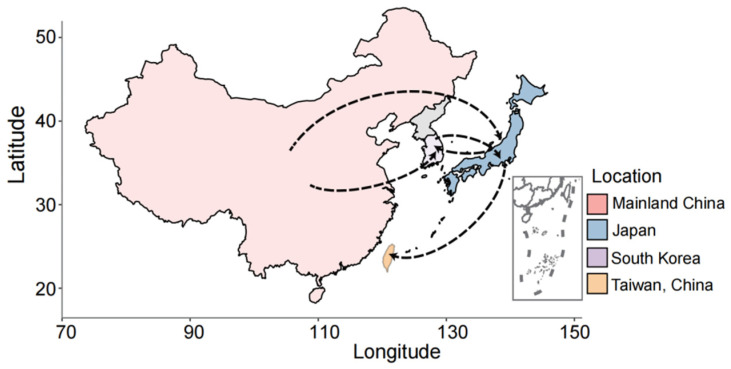
Schematic presentation of migration pathways of *M. oryzae* in localized regions of Asia. The arrows show the directions of immigration and emigration between the different locations.

Genetic differentiation between populations (FST) ranged from 0.088 to 0.237 (Figure 3A, Table S3) with a 20 kb window length and 2 kb steps, indicating moderate genetic differentiation. This result implies the likelihood of an *M. oryzae* migration occurring among the worldwide geographic populations. At the same time, the results from the phylogenetic network reconstruction exhibit reticulate relationships within the three clades, suggesting the occurrence of spatial migration (Figure 3B). In addition, the migration pathways of this pathogen that were inferred from the phylogeographic analysis revealed that migration events could be identified through the spatial diffusion of *M. oryzae* across Asia. Within the region of Asia, five major migration events were detected among China (including Taiwan Island and the mainland), Japan and South Korea (Figure 4). Mainland China acted as the primary source of gene migration, initially spreading to Japan and South Korea and subsequently extending to Taiwan Island via Japan. Additionally, bidirectional gene flows were observed between South Korea and Japan.

### 3.4. Demographic History of M. oryzae Populations

**Figure 5:**
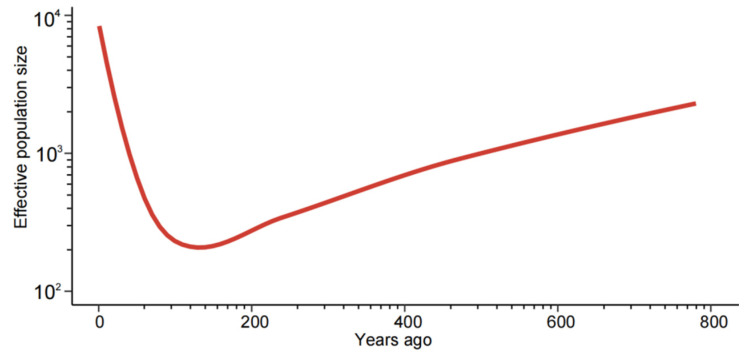
The demographic history of *M. oryzae*, illustrating the effective population sizes of the different geographical groups in Asia. The coordinates were logarithmically scaled. The colored lines represent the different geographical populations.

To investigate the demographic history of *M. oryzae*, effective population sizes were calculated using a pairwise sequentially Markovian coalescent (PSMC) model. As previously reported, rice-infecting isolates of *M. oryzae* evolved from grass-infecting isolates around 1000 years ago [23]. Therefore, effective population sizes were predicted for the last 800 years (Figure 5). Our findings indicated that *M. oryzae* underwent a striking population expansion in Asia approximately 100 years ago.

## 4. Discussion

For fungal pathogens of plants in agroecosystems, genetic diversity can reflect their capacity to adapt and survive under biotic and abiotic stresses [56,57]. This diversity is closely linked to their population origin and evolution, influencing their formation and maintenance [58,59]. In this study, the genetic diversity of the Chinese rice blast fungus population was shown to exhibit a continuously increasing trend since 1976. The primary factors influencing this trend may stem from the adaptation of *M. oryzae* to different geographical regions and rice varieties as a result of the improvement of rice cultivars, the expansion of cultivation areas, and the promotion of rice variety diversification in China, which likely led to an increase in genetic diversity over time. This suggests that Chinese populations could potentially serve as reservoirs of *M. oryzae* variants containing a greater array of novel genotypes, thereby enhancing their likelihood of becoming a source of diversity within Asian regions. Furthermore, among the three Asian regions studied, including Taiwan Island, mainland China, Japan and South Korea, the highest nucleotide diversity was observed in Chinese populations. This suggests that China serves as a center of genetic diversity for *M. oryzae* in Asia, as indicated by the microsatellite markers [24]. Based on its history of rice domestication, China is also deemed to be the center of origin for rice [60,61], meaning that *M. oryzae* would have accumulated more abundant genetic resources for adaptation and expansion across its host range. In conclusion, these results are consistent with the demographic history analysis in this study, which demonstrates a continuous expansion trend in the effective population size of *M. oryzae* in Asia over the past centuries.

For *M. oryzae* populations, the temporal dynamics of genetic diversity are often closely related to rice cultivation. According to the US Department of Agriculture (https://www.ers.usda.gov/data-products/rice-yearbook/, accessed on 11 May 2023), worldwide rice cultivation areas have expanded rapidly, increasing by 11% from 1960 to 2019. The main rice-planting acreage and production regions are concentrated in Asia, where *M. oryzae* populations have maintained a high level of genetic diversity. Factors contributing to this diversity include genomic diversification, genetic instability and high variation rates, such as the presence of abundant variable number tandem repeats (VNTRs) and transposable elements (TEs) [62,63,64]. The rapidly decreasing genetic diversity observed in the 2020s could potentially be attributed to insufficient sampling during this period. In addition, the frequent host-shifting and host range expansion of this pathogen in crops and weeds have historically contributed to its sustained high genetic diversity [37,65,66], along with the gene flow events between cereal- and grass-specific lineages [28]. However, the continually growing genetic diversity of *M. oryzae* poses a significant threat to rice production, as these pathogens with diversified genetic resources could more rapidly overcome resistant genes in rice. While this genetic diversity could provide important new insights into the development and deployment of resistant rice varieties in practice, under these scenarios, our findings underscore the need for greater attention to the effective monitoring and control of rice blast disease.

However, past hybridization events have imprinted on individual genome samples. In this study, the phylogenetic reticulate network and population differentiation indices indicated that incompatible events occurred within worldwide geographic populations, reflecting their harbored admixture status. In addition, three main clades were identified in worldwide isolates from the genetic structure analysis, with each clade containing individuals from different continents, suggesting that geographical location could not sufficiently account for population genetic differentiation in *M. oryzae.* Although geographical isolation plays an important role in shaping genetic divergence, leading to the geographic differentiation of many plant pathogen populations [67,68], the genetic differentiation of *M. oryzae* appears to be more vulnerable to its mating types [23]. While both MAT1-1 and MAT1-2 mating types have been identified in China [69], the individuals from China are distributed across three divergent clades. The population differentiation indices, as represented by FST values, demonstrated that African populations are genetically distant from other populations, aligning with previous findings that African populations maintain relatively distant genetic relationships with Asian populations and are predominantly distributed in MAT1-2 [19,34]. In terms of the population structures of *M. oryzae*, populations with only one mating type occupied the principal position, indicating that clonal lineages dominated by asexual reproduction were prevalent in natural populations. The species can be geographically separated into a series of less closely genetically related subpopulations according to population communication decreases [70] or gradual subpopulation increases following extinction and recolonization events. However, migration mediated by human activity and the transportation of infected seeds [33] has made plant pathogen populations deviate from their evolutionary routes through recombination. In this study, an incomplete separation signature was also detected in the SplitsTree analysis for the reticular structures in the clades, which contained samples from Asia, Africa, Europe and North America. Additionally, signals of genetic exchange were also detected among five continents, consistent with findings reported by Tharreau et al. [33] that indicate a global distribution of virulent genotypes. In prior research, Asia was regarded as a genetic reservoir, with Southeast Asia identified as the epicenter of origination and genetic diversity, as inferred from genetic diversity analysis [22,24]. This conclusion aligns with the putative origins of rice in southern China and northeast India [60,61,71,72]. As a seed-borne pathogen, *M. oryzae* can infect two major subspecies: *Oryzae sativa* subsp. *japonica* and *indica*. The *japonica* subspecies originated from rice domestication and then diverged into the *temperate* and *tropical japonica* subspecies, which progressively led to the formation of the *indica* subspecies. This implies a possibility for pathogen migration following the domestication and introduction of rice crops [71,73,74]. Within Asia, migration events were observed in South Korea, Japan and China. Given that China is considered the global origin of *M. oryzae*, it likely served as a foundational source for migration within Asia. Our results suggest that migration pathways originated from mainland China, initially extending to Japan and subsequently to Taiwan, China. These migration pathways have mainly been attributed to rice cultivars, as Japonica rice is preferred in both Japan and Taiwan, China. Furthermore, the limited trade and agricultural exchange between Taiwan Island and mainland China in recent decades could also have contributed to limited gene flow. Similarly, a reciprocal gene flow was observed between populations in Japan and South Korea, potentially influenced by frequent interstate trade between these two countries. A similar pattern of results has been observed in *Fusarium* head blight pathogens, where agricultural practices and human migration have been critical driving forces in shaping the population genetic structure of *Fusarium asiaticum* [75].

In summary, gene flows at different geographical scales have exerted a significant influence on the establishment of the genetic structure of *M. oryzae*. Understanding the spatiotemporal dynamics of population compositions following the success of introduced fungal pathogens is essential for combatting plant disease pandemics [76,77].

## Data Availability

Sequencing raw reads and assembled contigs are available from NCBI under BioProject ID: PRJNA1138824, BioSample: SAMN42726964 to SAMN42726985.

## Supplementary Materials

The following supporting information can be downloaded at https://www.mdpi.com/article/10.3390/jof10110739/s1, Table S1: Genome information for *M. oryzae* strains. Table S2: Cross-validation errors for different models. Table S3: Genetic differentiation among populations (*Fst*).
